# Supplementary material for: Science Education for the Youth (SEFTY): A Neuroscience Outreach Program for High School Students in Southern Nevada during the COVID-19 Pandemic
Source: eNeuro. 2024 Mar 29;11(4):ENEURO.0039-24.2024. doi: 10.1523/ENEURO.0039-24.2024 (PMC10999729; doi:10.1523/ENEURO.0039-24.2024)
Supplement: Figure 1-1 — An example of the SEFTY schedule and description of the lesson plan. Download Figure 1-1, DOCX file. [file eneuro-11-ENEURO.0039-24.2024-s001.docx]

**Extended Data Figure 1-1**

| **Schedule** | | |
| --- | --- | --- |
| Week 1 | Thursday  (10/6) | Introduction to the program, supplies, and metric system  Pre-evaluation assessment  Practice lab techniques  Transformation |
|  | Friday  (10/7) | Introduction to biology, the central dogma of biology, importance of genetics  Choose papers for Journal Club  How to approach reading peer-reviewed articles |
| Week 2 | Thursday  (10/13) | Sterile technique, inoculation, and media  Scavenger hunt around UNLV campus |
|  | Friday  (10/14) | Miniprep and nanodrop |
| Week 3 | Thrsday  (10/20) | Digestion and running digestion products on a gel |
|  | Friday  (10/21) | Discuss results, repeat experiment if needed |
| Week 4 | Thursday  (10/27) | Polymerase Chain Reaction  *Gattaca* movie |
|  | Friday (10/28) | PCR gel electrophoresis and imaging |
| Week 5 | Thursday  (11/3) | Introduction to cell culture (splitting cells) |
|  | Friday  (11/4) | View cells  Genetic disorder presentation  Work on Journal Club articles |
| Week 6 | Thursday  (11/10) | Transfection  *Human Nature* movie |
|  | Friday  (11/11) | View cells and image  Constructing a CV, crafting professional emails, cover sheets |
| Week 7 | Thursday  (11/17) | Introduction to histology and brain; view sections/image  Match regions to atlas of brain and match regions to disease |
|  | Friday  (11/18) | Review stations |
| No SEFTY this week - Thanksgiving break | | |
| Week 8 | Thursday  (12/1) | Journal Club Practice |
|  | Friday  (12/2) | Journal Club Presentations  Post-evaluation assessment |
